# A cross sectional analysis of eating habits and weight status of university students in urban Cameroon

**DOI:** 10.1186/s40795-017-0178-7

**Published:** 2017-07-11

**Authors:** Loveline L. Niba, Mary B. Atanga, Lifoter K. Navti

**Affiliations:** 1 Department of Biochemistry, Catholic University of Cameroon (CATUC), Bamenda, P.O. Box 782, Cameroon; 2 Nutrition and Health Research Group (NHRG), Bamenda, Cameroon; 3 Department of Nursing and Midwifery, University of Bamenda, P.O. Box 39, Bambili, Bamenda, Cameroon

**Keywords:** Overweight, Obesity, University students, Eating habits, Body mass index

## Abstract

**Background:**

The changeover from high school to university is characterized by the inability to make informed food choices and unhealthy eating habits. This study sets out to determine the prevalence of overweight/obesity, examine variations in dietary habits and assess the relationships between some dietary factors and overweight/obesity in university students.

**Methods:**

University students (*N* = 906, mean age 21.4 ± 2.1 years) that included 434 males and 472 females were recruited using a simple random sampling technique from six departments in two universities in a cross sectional study in the North West Region of Cameroon. Weight and height were measured and body mass index calculated. Eating habits and weekly consumption of selected food items were self-reported by the students using a pre-tested questionnaire.

**Results:**

The prevalence of overweight and obesity were 24.6% and 2.2% respectively. A majority (60.7%) of the students had less than three meals a day. Also, 53.4% ate fried foods, 46.0% had sweets/chocolates and 39.5% had sugar sweetened beverages twice or more times in a week. Skipping/rarely having breakfast (OR 1.8, 95% CI 1.2–2.9) and having snacks in-between meals three or more times a day (OR 2.2, 95% CI 1.4–5.5) were associated with overweight/obesity after controlling for confounding variables. In addition, skipping/rarely having breakfast (OR 2.2, 95% CI 1.3–3.5) independently predicted overweight/obesity in a model that included confounding variables and selected dietary behaviors.

**Conclusion:**

The unhealthy eating habits exhibited by students in this study is worrying. Qualitative studies need to be carried out in the future to identify determinants (of Cameroon ethnicity) of poor eating habits in university students.

## Background

The dramatic increase in the number of individuals with overweight/obesity in developing countries is concerning. A study among sub-Saharan African children and youth demonstrated that this increase is similar to that of developed countries [1], while another has indicated a higher relative increase (+65%) in developing countries than developed countries (+48%) [2]. In Cameroon, the numbers of individuals affected is on the rise and also remains a challenge. A survey that included participants, aged 15 years and over in four urban settings of Cameroon showed that 25% of men and almost half of the women were either overweight or obese [3]. Kiawi et al. [4] in another study in both rural and urban settings in the North West Region (NWR) of Cameroon estimated the prevalence of overweight and obesity to be 23.6% and 6.1% respectively. In addition, the Health of Populations in Transition Research Group in Cameroon revealed that in Bamenda (an urban setting and capital of the NWR), 33.6% and 23.3% of the study population were overweight and obese respectively [5].

There is enormous evidence indicating the contribution of a high body mass index (BMI ≥25 kg/m^2^) to some non-communicable diseases including type 2 diabetes [6], asthma [7], hypertension [8], cardiovascular diseases [9], stroke [10] and certain cancers [11]. A WHO report of 2014 indicates that the non-communicable diseases mentioned above accounted for approximately 31% of total deaths in Cameroon [12]. With respect to the above diseases, earlier reports in Cameroon also indicated that between 1994 and 2003, the age adjusted prevalence of overweight including obesity (BMI ≥25 kg/m^2^) increased significantly by 54% and 82% for men and women respectively [13, 14]. Over the same period, there was a 12.1% and 15.2% increase in age-adjusted prevalence of hypertension among women and men respectively [15], and a 10 fold increase in prevalence of diabetes among adults [16, 17]. However, these studies did not include the same subjects.

As young adults move from high school to an independent life in the university, they take full responsibility of their eating habits and most often, they have little or no guidance on how to make informed choices of the food they eat [18]. Many studies in different countries have been carried out to examine the effects of dietary factors on the weight status of young adults in the university. For instance, a US study revealed that there was a significant increase in consumption of alcohol and foods with a high fat content among university students [19]. Also, other studies have indicated a frequent intake of snacks (in-between meals) [20], sugar sweetened beverage [21] and smoking [22] among students. There is consistent evidence demonstrating the relationship between high consumption of sugar sweetened beverages and weight gain [23], but it is still a debate if reducing the intake of sugar sweetened beverages can lead to weight loss [24]. In addition, studies have reported that skipping meals, especially breakfast was common among Malaysian [25] and Italian university students [26]. Data on the eating habits in a university student population is scarce in Cameroon. Overweight and obesity is equally of much importance in this group because Silliman et al. [27] and Crombie et al. [28] have pointed out that during university life, students may acquire unhealthy nutritional habits that are linked to obesity, which may persist into adulthood.

This aim of this study is to determine the prevalence of overweight/obesity, examine variations in eating habits of the study population and assess the relationship between some dietary patterns (commonly observed among university students in previous studies) and overweight/obesity.

## Methods

### Subjects

This institution-based cross sectional analysis included a student sample from two universities (one public and one private) in Bamenda, NWR of Cameroon. Three departments were selected at random in each institution and lists that included first, second and third year students were obtained from each head of department. These lists were used to select students using a simple random sampling technique. An arrangement was made with each head of department and lecturers and the selected students were approached in their classroom immediately after lectures.

A total of 1310 students were selected and 1102 accepted to participate in the study. However, 196 students were dropped because of missing data, and the analysis finally included 906 students of mean age 21.4 ± 2.1 years (47.9% males and 52.1% females). The criteria used to exclude participants from the study included; pregnancy and breastfeeding.

### Ethical considerations

Ethical clearance was obtained from the institutional review board of the Catholic University of Cameroon (CATUC), Bamenda. Also, administrative clearance was obtained from the North West Regional Delegation for Public Health, Bamenda. The main objective of the study was explained to the participants and written informed consent obtained from each of them before data collection. They were told that their participation is voluntary.

### Anthropometry

The measurements were all campus-based (during the 2015/2016 academic year) and carried out by trained personnel, whose activities were in compliance with standard protocols. Standing height measurements (to the nearest 0.1 cm) were carried out with a portable stadiometer (Seca 213, Germany) without shoes. Body weight of participants (to the nearest 0.1 kg) was obtained using a portable digital scale (Omron BF 511, Japan) in light clothing and without shoes. The height and weight measurements were then used to calculate BMI (in kg/m^2^) as a measure of the weight status of participants. The WHO criteria on BMI was used to classify participants as underweight (BMI ≤ 18.5 kg/m^2^), healthy weight (BMI 18.5–24.9 kg/m^2^), overweight (BMI 25.0–29.9 kg/m^2^) and obese (BMI ≥ 30.0 kg/m^2^) [29].

### Food habits

As soon as anthropometric measurements were completed on each participant, they were given a pre-tested questionnaire and instructed on how to complete it. This questionnaire had been pre-tested in a group of 35 university students. In the absence of studies that examine dietary habits of university students in our setting, it was not possible to carry out any test of validity of the questionnaire. However, the questionnaire was developed by making use of previous studies that examined the dietary habits of university students in other countries [20, 21, 30]. The questionnaire collected self-reported information on age, gender, dietary behaviors/characteristics, and eating habits. The dietary characteristics section included questions on smoking status, alcohol intake, having a specific dietary regimen, presence of a chronic disease, frequency of eating at the university, number of meals in a day and the purchase of food. Smoking was categorized as: non-smoker, current smoke and ex-smoker. Alcohol intake was categorized as: never, rarely and 2 to 3 times per week. The response option for having a specific dietary regimen and presence of a chronic disease was ‘yes’ or ‘no’. Frequency of eating at the university was categorized as: 3 or more times a week, 1 to 2 times a week and never. The number of meals in a day was categorized as: one, two, three and more than three. Purchase of food was categorized as: food bought already cooked and raw food purchased and self-cooked.

The eating habits section included questions on the frequencies of having breakfast, eating while watching TV, snacks in-between meals in a day and fruit and vegetable intake. It also had a question on when participants stop eating. Having breakfast was categorized as: daily, 3 to 4 times a week, 1 to 2 times a week and skip/rarely. Eating while watching TV was categorized as: always, sometimes and never. Snacks in-between meals in a day was categorized as: once, twice, thrice and greater than three times. Fruit and vegetable consumption was categorized as: everyday, occasionally and never. When participants stop eating was categorized as: before fullness, at fullness and more than fullness. The participants also indicated the frequency of consumption of selected food items, which are popular among university students in Bamenda. These food items included: rice, bread, spaghetti, beans, fried Irish potatoes, fried plantain, sugar sweetened beverages, margarine, sweets/chocolate, cakes, meat/fish and eggs. The response option for the selected food items where: ≤ once/week, 2 to 4 times/week and ≥5times/week.

### Statistical analysis

Statistical analysis was carried out using IBM SPSS (version 21.0). The findings of the dietary behaviors/characteristics and assessment of dietary habits of participants were reported as numbers (%). The relationships between variables were reported as odds ratios with their corresponding confidence intervals. Normality was checked using Kolmogorov Smirnov (K – S) test. Also, a Chi square test was used to assess any significant differences in the consumption of selected food items between males and females. In addition, odds ratios were calculated using multivariable binary logistic regression analysis with overweight/obesity as dependent variable. The first step included models that assessed the relationship between each determinant and overweight/obesity and the corresponding odds ratios adjusted for age, gender, smoking, alcohol consumption, chronic disease, dietary regimen and type of institution (public and private). In the second step, odds ratios were adjusted for the above confounding variables and selected dietary behaviors (breakfast consumption, snacks in-between meals, eating while watching TV, fruits and vegetables consumption and when to stop eating). As a measure to ensure stability of the model, the standard errors were monitored so that they do not inflate by more than 10% when adding more variables in the model during the logistic regression analysis. Lastly, statistically significant differences were considered at *p* < 0.05.

### Sample size and power calculation

A major feature of the analysis in this study was the comparisons between males and females. R was used to compute the sample size with a power of 80%, using a two-sided test at the 5% level in order to detect a mean difference in BMI of 1.0 kg/m^2^ between males and females with a standard deviation of 4.1 [20]. The minimum sample required was 265 in each group, giving a total of 530.

### Results

The distribution of BMI and dietary characteristics of the investigated population is indicated in Table 1. The prevalence of overweight and obesity in this study were 24.6% and 2.2% respectively. More females were overweight (27.5%) than males (21.4%). However, obesity estimates were higher among males (2.5%) than females (1.9%). The majority (69.9%) of the students had a healthy weight.

**Table 1:** Gender distribution of dietary characteristics of a randomly selected sample of students at two universities in Bamenda, North West Region of Cameroon (*N* = 906)

Variables	Males		Females	
	*N* = 434		*N* = 472	
	*n*	%	*n*	%
Body mass index				
Underweight	13	3.0	17	3.6
Healthy weight	315	72.6	307	65.0
Overweight/obese	106	24.4	148	31.4
Smoking status				
Non smoker	393	90.5	461	97.7
Current smoker	25	5.8	11	2.3
Ex-smoker	16	3.7	0	0.0
Alcohol intake				
Never	116	26.7	164	34.7
Rarely	231	53.2	273	57.8
Two or more times a week	87	20.1	35	7.4
Dietary regimen				
Yes	30	6.9	42	8.9
No	404	93.1	430	91.1
Chronic disease				
Yes	28	6.5	26	5.5
No	406	93.5	446	94.5
Eating at the university				
Three or more times a week	154	35.5	150	32.2
One to two times a week	170	39.2	236	50.0
Never	110	25.3	84	17.8
Meals per day				
One	36	8.3	22	4.7
Two	217	50.0	275	58.3
Three	167	38.5	149	31.6
Greater than three	14	3.2	26	5.5
Purchase of food				
Ready-prepared meals	94	21.7	24	5.1
Raw food and self-cooked	340	78.3	448	94.9

Smoking was not common in our sample of students. However, more boys consumed alcohol than girls. A majority of the students ate at least once a week at the university with girls representing a higher proportion. Also, only 34.9% of study participants had three meals a day. Table 1 also shows that more boys purchased ready-prepared meals served in a restaurants than girls. Within the investigated population, majority (86.9%) of the students bought raw food and cooked it by themselves.

Table 2 compares the eating habits of males and females with respect to selected food items deemed to be popular among students in higher institutions of learning in Bamenda. The most frequently consumed food items (twice or more a week) by the students were bread followed by rice and then meat/fish, eggs and sugar sweetened beverages; while the least was spaghetti. There was a significant difference in consumption of rice and bread between males and females. More males ate rice and bread five or more times a week compared to females. However, there was no significant difference in the consumption of meat/fish and eggs by gender.

**Table 2:** Gender differences in the frequency of consumption of selected food items among a randomly selected sample of students at two universities in Bamenda, North West Region of Cameroon (*N* = 906)

Food items	Frequency	Males		Females		*p*-value
		*N* = 434		*N* = 472		
		n	%	n	%	
Rice	Less than once a week	50	11.5	94	19.9	≤ 0.001
	2 to 4 times a week	251	57.8	265	56.1	
	5 or more times a week	133	30.6	113	23.9	
Bread	Less than once a week	68	15.7	100	21.2	≤ 0.001
	2 to 4 times a week	217	50.0	259	54.9	
	5 or more times a week	149	34.3	113	23.9	
Spaghetti	Less than once a week	381	87.8	431	91.3	0.10
	2 to 4 times a week	32	7.4	30	6.4	
	5 or more times a week	21	4.8	11	2.3	
Beans	Less than once a week	332	76.7	389	82.4	0.05
	2 to 4 times a week	85	19.6	75	15.9	
	5 or more times a week	17	3.9	8	1.7	
Fried Irish potatoes	Less than once a week	287	66.1	267	56.6	0.01
	2 to 4 times a week	128	29.5	176	37.3	
	5 or more times a week	19	4.4	29	6.1	
Fried plantain	Less than once a week	376	86.8	399	84.5	0.28
	2 to 4 times a week	50	11.5	58	12.3	
	5 or more times a week	8	1.8	15	3.2	
Sugar sweetened beverages	Less than once a week	279	64.3	271	57.3	0.11
	2 to 4 times a week	125	28.8	161	34.2	
	5 or more times a week	30	6.9	40	8.5	
Margarine	Less than once a week	354	81.5	408	86.4	≤ 0.001
	2 to 4 times a week	52	12.0	60	12.7	
	5 or more times a week	28	6.5	4	0.8	
Sweets/ chocolate	Less than once a week	269	61.9	221	46.7	≤ 0.001
	2 to 4 times a week	124	28.6	190	40.3	
	5 or more times a week	41	9.4	61	13.0	
Cakes	Less than once a week	279	68.6	372	78.8	≤ 0.001
	2 to 4 times a week	109	25.2	91	19.3	
	5 or more times a week	27	6.2	9	1.9	
Meat/ fish	Less than once a week	143	32.9	145	30.7	0.08
	2 to 4 times a week	170	39.2	218	46.2	
	5 or more times a week	121	27.9	109	23.1	
Eggs	Less than once a week	148	34.1	178	37.7	0.33
	2 to 4 times a week	214	49.3	230	48.7	
	5 or more times a week	72	16.6	64	13.6	

Also, the consumption of margarine, sweets/chocolate and cakes were significantly different between males and females. More males (18.7%) ate margarine at least twice a week compared to females (14.8%). Similarly a higher proportion of males (31.5%) consumed cakes at least twice a week than females (21.4%). On the contrary, more than half of the female population (53.2%) reported consuming sweets/chocolate twice or more times a week compared to males (38.2%). A significant difference in the consumption of fried foods by gender was reported for fried Irish potatoes. More than half (53.4%) of the participants ate fried foods (fried Irish potatoes and plantain) twice or more times a week. The consumption of fried food five or more times a week was more common among females (9.5%) than males (6.2%).

Even though the consumption of sugar sweetened beverages (twice or more times a week) was common among the students (39.5%), there was no significant difference between males and females. The proportion of those who consumed beans at least twice a week was significantly higher for males (23.5%) than females (17.8%).

The relationships between overweight/obesity and potential factors is shown in Table 3. In the first set of models that controlled for age, gender, smoking, alcohol consumption, chronic disease, dietary regimen and type of institution, the habit of skipping/rarely having breakfast and having snacks (in-between meals) three or more times a day were significantly associated to overweight/obesity. However, eating while watching TV, fruit and vegetable consumption and when to stop eating were not significantly associated to overweight/obesity. Also, after controlling for the above confounding variables and selected dietary behaviors (breakfast consumption, snacks in-between meals, eating while watching TV, fruits and vegetables consumption and when to stop eating), skipping/rarely having breakfast was an independent predictor of overweight/obesity.

**Table 3:** Odds of overweight/obesity associated with selected dietary behaviors among a randomly selected sample of students at two universities in Bamenda, North West Region of Cameroon (*N* = 906)

Determinants	N	Overweight/obesity						
		Frequency (%)	Step 1 Adjusted OR^a^	(95% CI)	*p*-value	Step 2 Adjusted OR^b^	(95% CI)	*p*-value
Breakfast consumption								
Skip/rarely	242	23.6	1.8	(1.2–2.9)	0.01	2.2	(1.3–3.5)	0.002
One to two times a week	142	36.6	1.2	(0.8–1.9)	0.39	1.3	(0.8–2.1)	0.22
Three to four times a week	208	26.9	1.1	(0.7–1.6)	0.63	1.2	(0.8–1.8)	0.51
Daily	314	24.5	Ref			Ref		
Snacks (in-between meals)								
Greater than 3 times a day	68	20.5	2.2	(1.4–5.5)	0.04	2.2	(0.9–5.7)	0.10
Three times a day	36	33.3	1.1	(0.5–2.3)	0.77	0.9	(0.4–2.0)	0.81
Twice a day	134	23.9	1.4	(0.8–2.6)	0.27	1.5	(0.8–2.9)	0.22
Once a day	668	27.5	Ref			Ref		
Eating while watching TV								
Always	160	30.6	0.8	(0.5–1.4)	0.44	0.6	(0.4–1.1)	0.09
Sometimes	660	25.3	1.1	(0.6–2.0)	0.68	1.0	(0.5–1.8)	0.96
Never	86	30.2	Ref			Ref		
Fruits and vegetables consumption								
Never/occasionally	658	28.3	0.9	(0.6–1.3)	0.56	0.9	(0.5–1.3)	0.49
Everyday	248	26.2	Ref			Ref		
When to stop eating								
More than fullness	68	32.4	0.7	(0.4–1.1)	0.14	0.7	(0.3–1.4)	0.32
At fullness	670	26.1	0.7	(0.4–1.3)	0.27	0.7	(0.4–1.2)	0.21
Before fullness	168	26.8	Ref			Ref		

OR, odds ratio; CI, confidence interval

^a^Odds ratios have been adjusted for the following confounding variables: age, gender, smoking, chronic disease, having a dietary regimen and type of institution

^b^Odds ratios have been adjusted for the above confounding variables as well as breakfast consumption, snacks in-between meals, eating while watching TV, fruits and vegetables consumption and when to stop eating

### Discussion

This study was designed to determine the prevalence of overweight and obesity, examine the differences in dietary habits of the study population and also assess the relationship between dietary factors and overweight/obesity. It has shown that more than a quarter of the university students were overweight/obese. Similar findings were reported by other studies in Malaysia [31], Jordan [32], Iran [33] and Saudi Arabia [34]. Also, higher prevalence estimates have been reported in the US [35], Kuwait [36] and United Arab Emirates [37], while a Nigerian study reported lower estimates [38]. These differences could be as a result of socio-cultural disparities, which may affect the lifestyle, food habits and health behavior of the students [33], differences in sampling techniques, sample sizes of studies and to some extent on how the data were collected. For instance, height and weight in some studies were measured, while in others these variables were self-reported.

The eating habits of having less than three meals a day and frequent consumption of fried foods, sweets/chocolates, sugar sweetened beverages and cakes observed in this study could be as a result of convenience and the availability of these items along the streets and in university canteens. The less frequent consumption of fruits and vegetables among the students could be explained by the high prices especially in the dry season. Also, the consumption of raw vegetables and fruits alongside main meals is not a common practice in the student population.

This unhealthy picture is typical of a university student population as demonstrated in previous studies which identified poor dietary behavior among university student samples in Jordan [32] and Nigeria [39]. In addition, a report by Al-Rethaiaa et al. [40] revealed that most of the students had two meals a day, with frequent consumption of fried foods and snacks, and less frequent consumption of fruits and vegetables. However, a study in Finland showed that most university students adhere to the dietary guidelines of consumption of cakes, fruits, vegetables, fast foods and sugar sweetened beverages [30].

Some studies had identified potential determinants that could explain the poor eating habits of university students. For instance, a recent study that included a student sample from 40 German universities identified high food prices, lack of time and absence of healthy foods in the university canteen as factors that influence healthy eating [41]. Even though there is no documented evidence, a majority of Cameroon university students do not work, and they rely on their parents’ support throughout university studies. Those from a low income background might not be able to afford three healthy meals in a day. In addition, a majority of students spend most of their time on campus and have limited time to prepare a healthy meal for themselves, especially during examination periods [42] and thus rely on confectioneries that form the bulk of foods available in university canteens.

Nutrition knowledge had been identified as an additional factor that could explain poor eating habits among university students [42]. This study did not assess nutrition knowledge. However, a US study revealed that only 4% of a university student population had good nutritional knowledge [43]. Thus a limited nutrition knowledge could explain to some extent the unhealthy dietary habits observed in this study.

However, the last two studies were each carried out in one university and there could have been some degree of selection bias as the studies made use of volunteers.

The high frequency of consumption of rice, bread, eggs and meat/fish especially among the males observed in this study could be because these food items are more convenient/require minimal preparation. The consumption of these food items were also common among students in Saudi Arabia [21]. However, this study included only female subjects from one university. Eggs was also frequently consumed because it is easily affordable and fried eggs with bread or fried potatoes/plantains or spaghetti are popular combinations eaten by the students. Additionally, rice and stew (including meat, fish or chicken and vegetable) is another popular dish among university students. Beans is usually eaten with rice or fried plantains. However, the low consumption of beans by students in this study could be because beans is bought raw and takes a longer time prepare.

Skipping/rarely having breakfast and having snacks (in-between meals) three or more times in a day were associated with overweight/obesity. Evidence has shown that when people skip breakfast, there is a tendency that their snacks intake will be higher during the day, which could lead to a positive energy balance and weight gain [44]. However, a study indicated no relationship between frequency of breakfast intake and overweight/obesity [45] and the contribution of snacks to a higher BMI is still a subject of debate [41].

This study had limitations. Some subjects had to be dropped because of missing data. However, there were no significant differences in anthropometric variables between those dropped and those retained in the study. Also, the standard errors might have been underestimated as a result of the fact that a simple random sampling technique was used to recruit participants. The possibility of selection bias cannot be ruled out because students participated on a voluntary basis. The information on dietary habits and weekly consumption of foods were self-reported and could have been subjected to recall bias. Our study included participants from one out of the ten administrative regions of the country. Thus, the findings may not be representative of the university student population of the whole country. Elements of causality cannot be established as a result of the cross sectional nature of the study. Additionally, physical activity and calorie intake, which are important determinants of overweight/obesity were not assessed. Nevertheless, this study had some strengths. It included students from two universities as opposed to one university as reported in other studies [20, 21, 25, 26, 30, 32, 33, 37, 42]. The study population included a mix of students from different socioeconomic backgrounds because it included students from both a state and a private university. In Cameroon, students in private institutions are usually of a higher socioeconomic background compared to those in state institutions because of the higher tuition fees in private institutions. This could have eliminated the confounding effect of socioeconomic status to a certain degree.

## Conclusion

More than a quarter of the study participants were overweight/obese, and majority had a healthy weight. This study has demonstrated that students who skip/rarely have breakfast tend to have higher odds of overweight/obesity. It has also revealed that majority of the students had less than three meals a day and chose the unhealthy food options. In the future, it is important for studies to consider the use of a qualitative approach to find out the determinants (of Cameroon ethnicity) that affect eating habits of university students.

## Abbreviations

BMI: Body mass index
NMR: North West Region
TV: Television
WHO: World Health Organization
